# Application of electrically evoked auditory brainstem responses in cochlear reimplantation

**DOI:** 10.3389/fnins.2026.1915543

**Published:** 2026-09-22

**Authors:** Ying Jin, Qingling Bi, Yong Lv, Zhongyan Chen, Yang Liu, Jianfeng Liu, Yan Li, Wenjing Yang, Xiaoyu Xu, Yuan Li, Zuomin Wang

**Affiliations:** 1 Department of Stomatology, Beijing Chao-Yang Hospital, Capital Medical University, Beijing, China; 2 Department of Otolaryngology-Head & Neck Surgery, China-Japan Friendship Hospital, Beijing, China

**Keywords:** cochlear implantation, EABR, ossification, performance, reimplantation

## Abstract

**Objectives:**

To evaluate the role of electrically evoked auditory brainstem responses (EABRs) in assessing auditory pathway integrity and predicting auditory and speech outcomes in patients undergoing cochlear reimplantation (CRI), especially those with cochlear ossification or complex anatomy.

**Methods:**

A retrospective analysis was conducted of 40 CRI patients (12 ossification, 28 non-ossification) from 2019 to 2024. Intraoperative and postoperative EABR assessments were performed to evaluate neural responses and guide cochleostomy. Parameters included the elicitation rate, V-wave amplitude, threshold, and Gibson score. Auditory–speech performance was measured by the Categories of Auditory Performance and Speech Intelligibility Rating scales, and word-recognition scores. Regression analyses identified predictors of postoperative outcomes.

**Results:**

The EABR elicitation rate was 100% in non-ossification cases and 80% in ossification cases. Gibson scores were higher, and V-wave thresholds lower, in non-ossification ears (*p* < 0.05). Latencies and amplitudes did not differ significantly. Auditory–speech outcomes were superior in non-ossification patients. Implantation age, cochlear ossification, and middle-electrode Gibson score significantly predicted postoperative performance; middle-electrode Gibson score correlated most strongly with the disyllabic word recognition rate (R^2^ = 0.406, *p* = 0.0007).

**Conclusions:**

EABRs reliably assess auditory pathway integrity, inform surgical guidance, and predict postoperative auditory–speech outcomes in CRI, especially in ossified or anatomically complex cochleae.

**Significance:**

The findings support the use of EABRs as a clinical adjunct to improve surgical precision and functional outcomes in challenging CRI cases.

## 1 Introduction

Cochlear implants (CIs) are the standard treatment for individuals with bilateral severe-to-profound sensorineural hearing loss (House, 1987; Mudry and Mills, 2013). Since the first implantation in 1961, CIs have been widely adopted worldwide. However, the limited lifespan of electronic devices makes them susceptible to various malfunctions that may necessitate reimplantation in both the short and long term. It has been estimated that children with hearing impairment may require two to three CI devices over their lifetime.

The first reported cochlear reimplantation (CRI) procedure was performed by Hochmair-Desoyer and Burian in 1985 in two patients using Vienna CIs (Hochmair-Desoyer and Burian, 1985). Reported reimplantation rates across studies are currently 0.5%–30%, depending on the patient population and the duration of follow-up (Asghari et al., 2024; Smetak et al., 2023). As the number of CI recipients continues to increase, the incidence of CRI—primarily driven by device failure, medical complications, and, to a lesser extent, technological upgrades—is also expected to rise steadily. The indications for reimplantation can be categorized as device-related (hard failure or soft failure) or medical, with device-related complications remaining the most common cause (Chen et al., 2024; Kim et al., 2023).

Overall, CRI is considered a safe procedure, and most studies have demonstrated that it can preserve or even improve auditory and speech performance. Nevertheless, approximately 15%–16% of children experience deterioration in outcomes after reimplantation (Blanc et al., 2022). Factors, such as cochleostomy-related trauma, foreign body reactions to the electrode array, and intracochlear inflammatory responses are associated with cochlear ossification and fibrosis following initial implantation (Danielian et al., 2021), potentially complicating electrode reinsertion. In addition, these newly formed tissues may disrupt the electrode–neural interface, thereby negatively affecting auditory and speech perception and potentially contributing to device malfunction and soft failure (Donati et al., 2023; Kamakura and Nadol, 2016).

In addition to intracochlear tissue formation, other proposed causes of postoperative performance deterioration include scala vestibuli insertion of the electrode array, initial diagnostic inaccuracies, soft tissue collapse within the scala tympani that impedes proper reinsertion, prolonged periods of device non-use before reimplantation, and poor adherence to postoperative speech rehabilitation programs (Blanc et al., 2022).

The electrically evoked auditory brainstem response (EABR) is a diagnostic modality analogous to acoustic auditory brainstem response testing, but it uses direct electrical stimulation of the cochlea to assess auditory brainstem function. EABRs can be elicited either extracochlearly, such as at the round window niche (Causon et al., 2019), or intracochlearly through a CI (Firszt et al., 2002). Because post-implantation outcomes may correlate with the number and functional integrity of surviving auditory nerve fibers, EABR thresholds have shown prognostic associations with performance in CI users, particularly within the low-to-mid frequency range (Chen et al., 2025). Therefore, EABRs may also have value in predicting postoperative outcomes following CRI.

Furthermore, the surgical technique used during the initial implantation and the intraoperative conditions encountered may introduce uncertainty during reimplantation, particularly in complex cases such as severe cochlear ossification. We, therefore, hypothesized that preoperative or intraoperative assessment of neural pathway integrity using EABRs could facilitate successful reimplantation and provide predictive information regarding postoperative outcomes.

In this study, we retrospectively analyzed the surgical, electrophysiological, and postoperative outcomes of 40 CRI cases treated at the China–Japan Friendship Hospital. We compared EABR findings with postoperative speech rehabilitation outcomes in patients with normal cochlear morphology and those with cochlear ossification or inner-ear deformities. The study had two primary objectives: to characterize EABR waveform patterns in patients with cochlear ossification or abnormal cochlear anatomy, and to identify electrophysiological parameters that may predict auditory and speech rehabilitation outcomes following CRI.

## 2 Materials and methods

### 2.1 Subjects

A retrospective study was conducted of 40 CRI cases with an EABR electrophysiological assessment that was performed at our center between January 2019 and September 2024. Among these, one case was identified from a cohort of 1,123 patients who underwent initial cochlear implantation at our center between March 2007 and September 2024. The remaining 39 cases underwent initial implantation at other domestic cochlear implant centers and were subsequently referred to our institution for reimplantation surgery. The experimental protocol and study design were reviewed and approved by the Ethics Committee of the China–Japan Friendship Hospital. Written informed consent was obtained from all participants or their legal guardians before study initiation.

### 2.2 Clinical data collection

The clinical data collected from the patients included the timing of the initial cochlear implantation, the timing of CRI, and the indications for reimplantation. Surgical records from the initial implantation, preoperative temporal bone computed tomography (CT) scans, and audiological evaluations performed at 3 months after CRI were reviewed. Manufacturer-specific device integrity test reports, as well as device programming and rehabilitation records, were also collected.

The indications for CRI were categorized as device failure or medical complications. Hard failure was defined as the complete absence of auditory stimulation from the CI. Cases with normal device integrity testing but persistent complaints, such as dizziness or declining auditory performance, were classified as soft failure. Medical complications included wound or flap-related problems, meningitis, implant extrusion, magnet migration, improper initial implantation, and facial-nerve stimulation.

### 2.3 Evaluation of cochlear ossification and fibrosis

Cochlear ossification can be confirmed by CT and magnetic resonance imaging (MRI). Because MRI was contraindicated or infeasible for patients with CRI, the diagnosis of cochlear ossification and fibrosis relied primarily on preoperative temporal bone CT and intraoperative exploration.

(1). Temporal bone CT findings: narrowing of the bony cochlear duct, irregularities of the inner wall of the cochlear duct, and bony hyperplasia around the cochleostomy site.

(2). Intraoperative observations: bony hyperplasia around the cochleostomy site, narrowing of the cochlear duct, resistance encountered during electrode extraction and reinsertion, and partial electrode extrusion surrounded by dense fibrous tissue causing resistance during extraction. In such cases, enlargement of the cochleostomy and removal of fibrous tissue within the basal turn of the cochlea were required to facilitate insertion of the new electrode array.

### 2.4 Assessment of speech and listening ability

Auditory and speech performance were evaluated at 3 months after CRI using Mandarin Speech Perception word lists, with outcomes quantified by monosyllabic and disyllabic word-recognition scores. Stimuli were presented in the sound field at 65 dBA through a single loudspeaker, with the subject seated 1 m from the loudspeaker and directly facing it.

In addition, standardized Chinese versions of the Categories of Auditory Performance (CAP) and Speech Intelligibility Rating (SIR) scales were administered to evaluate postoperative auditory behavioral responses and speech intelligibility. The CAP scale consists of nine hierarchical levels (0–9), whereas the SIR scale comprises five levels (1–5).

### 2.5 EABR procedures

#### 2.5.1 EABR recording

Evoked auditory brainstem response evoked via the round window membrane: EABR recordings were obtained using Neuro-Audio NET1.0.103.3 (Neurosoft, Ivanovo, Russia). Needle electrodes were used as the recording, ground, and reference electrodes and were placed at the midline forehead, between the eyebrows, and the contralateral shoulder, respectively. Electrical stimulation was generated using an electromyography external electrical stimulator (Neurosoft, Ivanovo, Russia). A facial-nerve stimulation probe (Medtronic, Minneapolis, MN, USA) was used as the stimulating positive electrode. A stainless-steel needle electrode served as the reference electrode and was positioned over the ipsilateral external occipital protuberance. The initial stimulation intensity for EABR recording was set at 2.0 mA. Each stimulation intensity was tested twice, and the two waveforms were superimposed before proceeding to the next intensity level. The maximum stimulation intensity was 3.0 mA.

Postoperative EABRs: The recording electrodes and recording parameters used for postoperative EABRs were identical to those described above. Alternating biphasic pulses in monopolar mode (MP 1 + 2), with a pulse width of 50 μs (MED-EL devices [MED-EL GmbH, Innsbruck, Austria] set at 37 CU]; Nurotron devices [Nurotron Biotechnology Co., Ltd., Hangzhou, China] set at 50 CU) and an interphase gap of 5 μs, were used as stimuli. Stimuli were delivered at a rate of 30 Hz through the Nurotron Processor Interface and MED-EL, controlled by dedicated software (NuroSound 1.1.0; Nurotron Biotechnology). To explore the EABR characteristics from high- to low-frequency stimulation sites, EABRs were obtained by applying electric stimulation at one apical, one middle, and one basal electrode (i.e., electrodes 1, 11, and 22) representing low, middle, and high frequencies, respectively.

The EABR output signal was filtered online using a band-pass filter of 0.1–3 kHz and averaged over 512 sweeps at each stimulation level within a 15-ms recording window. Electrical stimulation consisted of alternating wave pulses with a duration of 100 μs delivered at a rate of 21 Hz. Electrode impedance was maintained at below 1 kΩ.

#### 2.5.2 Surgical procedures

Following mastoidectomy and removal of neo-osteogenic and hyperplastic fibrotic tissue within the facial recess, posterior tympanotomy was performed to expose the original electrode array. To minimize interference from muscle activity during EABR recording, patients received cisatracurium at a dose of 0.5 mg/kg according to body weight. After removal of the original electrode array from the cochlea, the stimulation probe was positioned at the original cochlear fenestration site for EABR recording.

#### 2.5.3 EABR data processing and analysis

All EABR analyses were independently performed by two experienced professionals, including an otolaryngologist and an audiologist.

EABR evoked via the round window membrane: Repeatable and stable EABR waveforms were evoked at the maximum stimulation level.

Postoperative EABRs: Routine parameters were evaluated according to the Gibson scoring system, in which a maximum score of 3 indicates the presence of clearly identifiable waves II, III, and IV–V, with a wave V amplitude exceeding 0.5 μV (Chen et al., 2024). The latencies of eIII and eV at 20 CL above threshold were recorded. Two threshold definitions were adopted. The EABR threshold was defined as the stimulation intensity immediately before the waveform amplitude decreased below 0.1 μV or before stable, reproducible, high-quality wave V responses could no longer be identified. The 0.5-μV threshold was defined as the stimulation intensity immediately before the waveform amplitude decreased below 0.5 μV. The response rate was calculated by dividing the number of patients with positive EABRs by the total number of subjects included in the study.

### 2.6 Statistical analysis

Statistical analyses were performed to evaluate differences in study variables between groups with and without cochlear ossification. Normally distributed continuous variables were analyzed using independent-samples *t*-tests, whereas non-normally distributed continuous variables were analyzed using the Wilcoxon signed-rank test. Categorical variables were compared using the chi-square test or Fisher’s exact test, as appropriate. Data normality was assessed using the Shapiro-Wilk test.

To investigate the relationship between EABR parameters and postoperative outcomes, univariate analyses were performed using CAP, SIR, and monosyllabic and disyllabic word-recognition scores as dependent variables, while EABR parameters and age at implantation were treated as independent variables. Age at implantation was identified as a significant factor in the univariate analysis. Therefore, multivariate analyses were subsequently conducted with age at implantation included as a covariate to assess the influence of EABR parameters on CAP, SIR, and monosyllabic and disyllabic word-recognition scores. Adjusted odds ratios and 95% confidence intervals were calculated to quantify associations.

All statistical analyses were performed using R software version 4.2.2 (R Foundation for Statistical Computing, Vienna, Austria). A *p*-value of <0.05 was considered statistically significant.

## 3 Results

In total, 12 patients were identified as having cochlear ossification based on preoperative CT imaging and intraoperative findings. Among these, one case (No. 37) was associated with a medical complication. This patient had developed localized skin infection and necrosis 3 years after the initial cochlear implantation, which eventually resulted in flap breakdown and device exposure. Following debridement and removal of the receiver–stimulator, the patient underwent CRI 3 months later. During surgery, ossification of the basal turn of the cochlea was observed, and complete electrode insertion was achieved after partial removal of hyperplastic bone and fibrous tissue. The remaining 11 cases underwent reimplantation because of device failure, including nine cases of soft failure and two cases of hard failure. Detailed clinical characteristics of these 12 patients are summarized in Figure 1 and Table 1.

**FIGURE 1:**
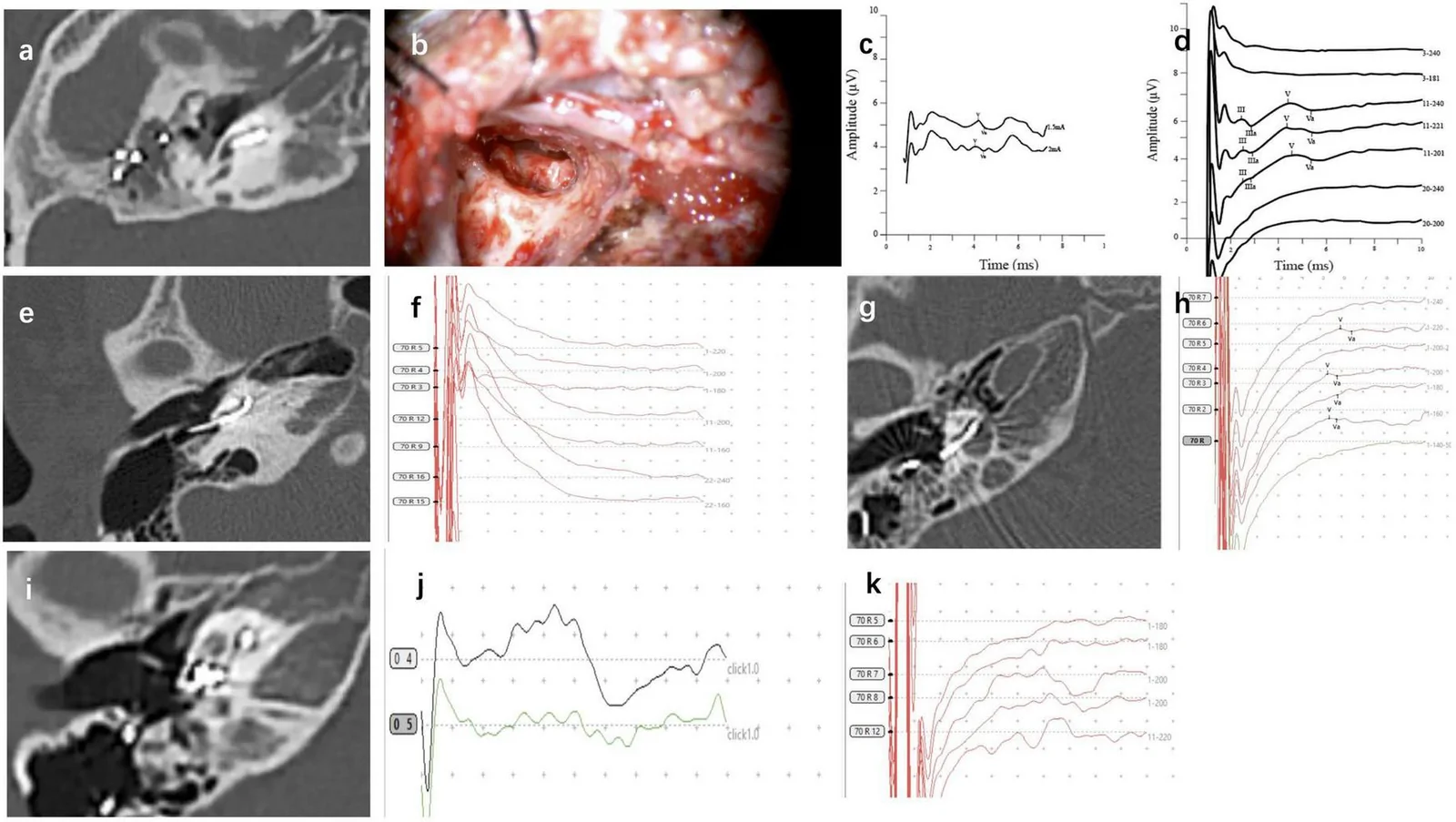
**(a)** Temporal computed tomography (CT) revealed cochlear ossification in the basal turn, consistent with the intraoperative finding of substantial ossification obstructing the original cochleotomy site during cochlear reimplantation (CRI) surgery **(b)**. Evoked auditory brainstem responses (EABR) recordings obtained via the round window membrane demonstrated repeatable and distinct wave V responses **(c)**, further confirming that the pore served as the appropriate insertion pathway. Postoperative EABR showed favorable waveforms in the middle electrodes **(d)**. **(e–k)** Correspond to Patients 8 and 10, respectively. Both patients exhibited cochlear ossification on CT scans and flat EABR responses, with Waves III and V not being identified. **(j,k)** Correspond to Patient 7, in whom cochlear ossification was identified in the basal turn. EABR recorded via the round window membrane demonstrated unfavorable waveforms, and only partial implantation was achieved, accompanied by poor postoperative EABR responses.

**TABLE 1:** Detailed clinical characteristics of 12 patients with cochlea ossification.

Subject no.	Sex	Side	Mode	Etiology	CI type	Sound processor			CRI reason	Electrode insertion	Pathology	Radiology
1	M	R	B	NA	CS-10A	NSP-60B	4.8	2.2	Soft	Complete	–	–
2	M	R	B	NA	CS-10A (TM)	Voyager	13.8	6.6	Soft	Complete	Acute and chronic inflammatory cell infiltration within the cochlear fibrous tissue, accompanied by a multinucleated giant cell reaction	Basal cochlear ossification
3	F	R	M	NA	CS-10A (TM)	Enduro	10.3	5.8	Soft	Complete	NA	Basal cochlear ossification
4	M	R	M	NA	CS-20A (TM)	Voyager	7.3	2.4	Soft	Complete	NA	Basal cochlear ossification
5	F	L	M	NA	CS-10A (TM)	Enduro	–	–	Soft	Complete	Intracochlear tissue revealed scant fibrous connective tissue with focal calcifications, and foreign-body giant cell reactions	Basal cochlear ossification; large bony fragment
6	M	R	B	EYA1 chr8:72127661; CNC narrowing	CS-10A	Enduro	9.3	1.3	Soft	Partial/18	Chronic inflammation of the fibrous connective tissue, accompanied by deposition of cholesterol crystals	Pericochlear bone hyperplasia
7	F	R	M	NA	CS-10A	Enduro	8	4.2	Soft	Partial/8	–	–
8	M	R	M	NA	CS-20A	Voyager	16.75	9.9	Soft	Complete	–	–
9	M	L	B	Auditory neuropathy spectrum disorder	Helix adv.1j/Q30	Harmony	4	1	Medical	Complete	Granulation tissue with multinucleated giant cells, fibrous encapsulation of the electrode, and chronic inflammatory infiltrates (lymphocytes, plasma cells, and eosinophils)	–
10	M	R	B	IP-III; POU3F4, CHM	CS-10A	NSP-60C	8	6	Soft	Partial/19	–	–
11	M	L	M	NA	CS-10A	NSP-60C	19	8.5	Hard	Complete	–	–
12	M	R	B	LVAS, Mondini	CS-10A (TM)	Voyager	14	5	Hard	Complete	–	–

The non-ossification group included 28 patients. Among them, seven cases were attributed to hard failure, 20 to soft failure, and 1 to a medical complication. This patient (No. 26) developed recurrent meningitis due to a meningeal defect 11 years after the initial implantation. The defect was surgically repaired, and CRI was successfully performed 3 months later without evidence of cochlear ossification. The EABR waveforms recorded from three electrode locations in this patient demonstrated good waveform differentiation.

Table 2 summarizes the key baseline characteristics of both groups, including age at reimplantation, duration of deafness, etiology, and electrode type. As shown in Table 2, there were no significant differences in age at reimplantation, duration of deafness, etiology, or electrode type between the ossification and non-ossification groups, which further supports the reliability and validity of our findings.

**TABLE 2:** Baseline comparison between the non-ossification and ossification groups.

Region/variable	Non-ossification group (*n* = 28)	Ossification group> (*n* = 12)	Total (*N* = 40)	t/χ^2^/Z	*P*-value
Testing age, y				185	0.342
Median [Q1, Q3]	13 [7, 16.60]	9 [7, 14.00]	11 [7, 16.20]	–	–
Implantation age/y				167	0.706
Median [Q1, Q3]	5 [2, 9.15]	5 [2, 6.60]	5 [2, 8.30]	–	–
Sex				–	0.725
F	9 (32.14%)	3 (25.00%)	12 (30.00%)	–	–
M	19 (67.86%)	9 (75.00%)	28 (70.00%)	–	–
Implanted ear					0.725
L	9 (32.14%)	3 (25.00%)	12 (30.00%)	–	–
R	19 (67.86%)	9 (75.00%)	28 (70.00%)	–	–
Mode				–	0.728
B	11 (39.29%)	6 (50.00%)	17 (42.50%)	–	–
M	17 (60.71%)	6(50.00%)	23(57.50%)	–	–
Etiology				–	0.489
Aminoglycoside antibiotics	1 (3.57%)	0 (0.00%)	1 (2.50%)	–	–
EYA1	0 (0.00%)	1 (8.33%)	1 (2.50%)	–	–
GJB2	6 (21.43%)	0 (0.00%)	6 (15.00%)	–	–
IP-III, POU3F4	0 (0.00%)	1 (8.33%)	1 (2.50%)	–	–
LVAS	3 (10.71%)	1 (3.57%)	4 (14.29%)	–	–
PTPN11	1 (3.57%)	0 (0.00%)	1 (2.50%)	–	–
Hypoplastic cochlear nerve	0 (0.00%)	1 (8.33%)	1 (2.50%)	–	–
NA	17 (60.71%)	8 (66.67%)	25 (62.50%)	–	–
Type of RCI				–	0.454
CS-10A	11 (39.29%)	5(41.67%)	16 (40.00%)	–	–
CS-10A (TM)	12 (42.86%)	4 (33.33%)	16 (40.00%)	–	–
CS-20A	0 (0.00%)	1 (8.33%)	1 (2.50%)	–	–
CS-20A (TM)	4 (14.29%)	1 (8.33%)	5 (12.50%)	–	–
Helix adv.1j/ Q30	0 (0.00%)	1 (8.33%)	1 (2.50%)	–	–
SONATA TI100+F28	1 (3.57%)	0 (0.00%)	1 (2.50%)	–	–
Type of sound processor				–	0.805
Enduro	8 (28.57%)	4 (33.33%)	12 (30.00%)	–	–
NSP-60B	4 (14.29%)	1 (8.33%)	5 (12.50%)	–	–
NSP-60C	4 (14.29%)	2 (16.67%)	6 (15.00%)	–	–
Sonata/Opus 2	1 (3.57%)	0 (0.00%)	1 (2.50%)	–	–
Voyager	11 (39.29%)	4 (33.33%)	15 (37.50%)	–	–
Advanced Bionics	0 (0.00%)	1 (8.33%)	1 (2.50%)	–	–

RCI auditory performance was available for 35 of 40 patients (87.5%). In the non-ossification group, the mean CRI CAP and SIR scores were 6.19 ± 1.5 and 3.77 ± 0.95, respectively, and the mean monosyllabic and disyllabic word-recognition rates were 71.2 ± 25.5% and 73.6 ± 24.8%, respectively. In the ossification group, the mean pre-failure CAP and SIR scores were 4.17 ± 1.34 and 2.25 ± 0.87, respectively, and the mean monosyllabic and disyllabic word-recognition rates were 38.0 ± 23.5% and 41.5 ± 24.7%, respectively. All patients with available data obtained a measurable auditory benefit from their initial implant prior to failure, confirming that the auditory pathway was functional before reimplantation in these cases.

### 3.1 Intraoperative EABR results

Intraoperative EABR recordings via the round window niche were successfully obtained in 11 patients during CRI surgery.

### 3.2 Comparison of postoperative EABR parameters and auditory–speech performance between the two groups

In the non-ossification group, 27 of 28 patients demonstrated well-differentiated EABR waveforms, yielding an elicitation rate of 100%; one patient (No. 28) did not undergo EABR testing. In the ossification group, two patients did not undergo EABR testing, and one patient had EABR recordings obtained only from the apical electrode. Among the remaining patients, two failed to demonstrate reproducible EABR waveforms across all three electrode positions, resulting in an elicitation rate of 80% (*n* = 8/10).

Gibson scores recorded from the apical, middle, and basal electrodes were significantly higher in the non-ossification group than in the ossification group (apical: *p* = 0.002; middle: *p* = 0.002; basal: *p* = 0.0001) (Figure 2). Analysis of Wave III and Wave V latencies revealed no significant differences between the two groups across all three electrode positions. However, V-wave thresholds, including both the 0.5-μV threshold (Figures 3a, b) and the disappearance threshold (Figures 3c, d), were significantly lower at the middle and basal electrodes in the non-ossification group compared with the ossification group (*p*-values are presented in Table 3). No significant differences in V-wave amplitudes were observed between the two groups.

**FIGURE 2:**
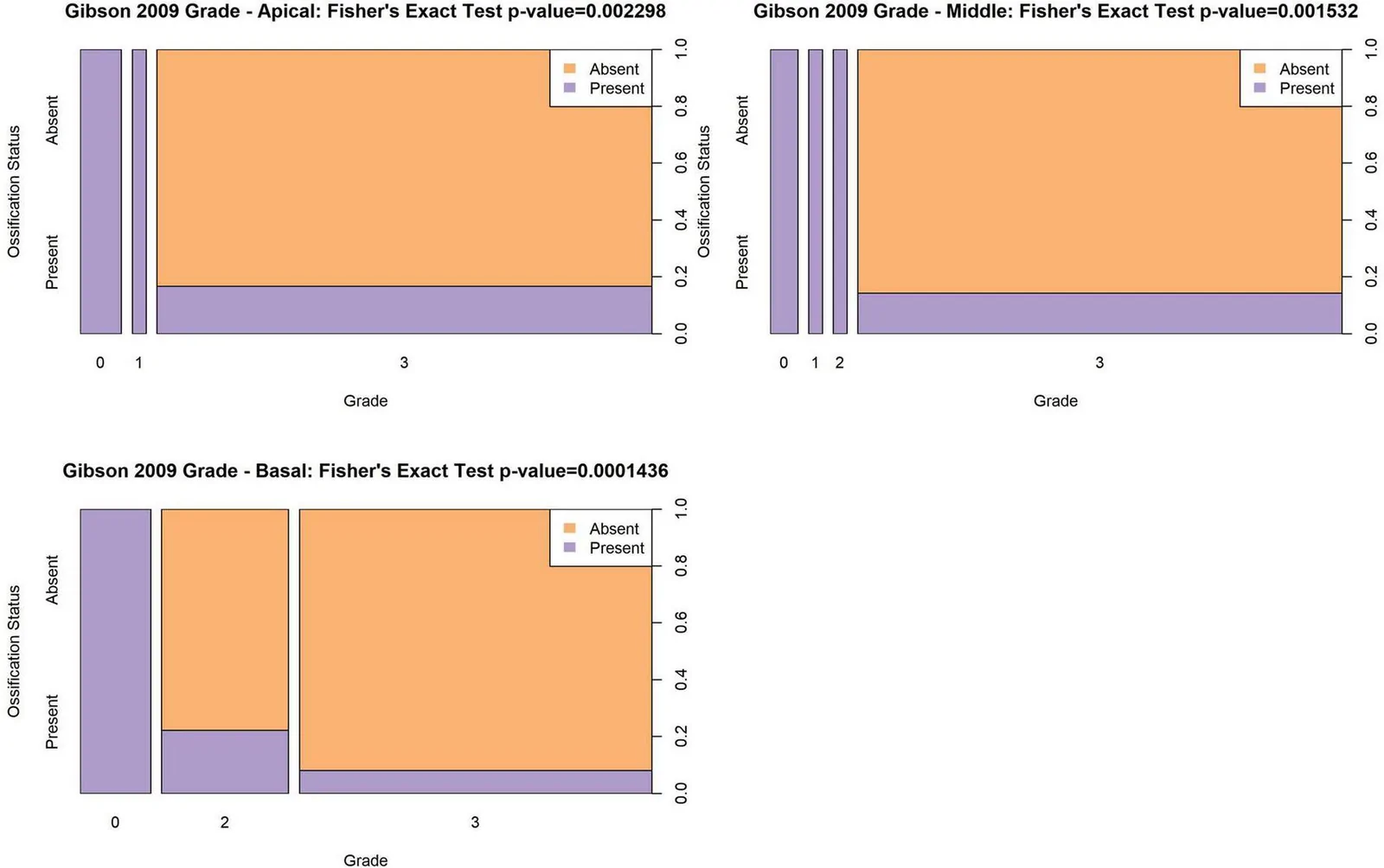
Gibson scores recorded from the apical, middle, and basal electrodes were significantly higher in the non-ossification group than in the ossification group.

**FIGURE 3:**
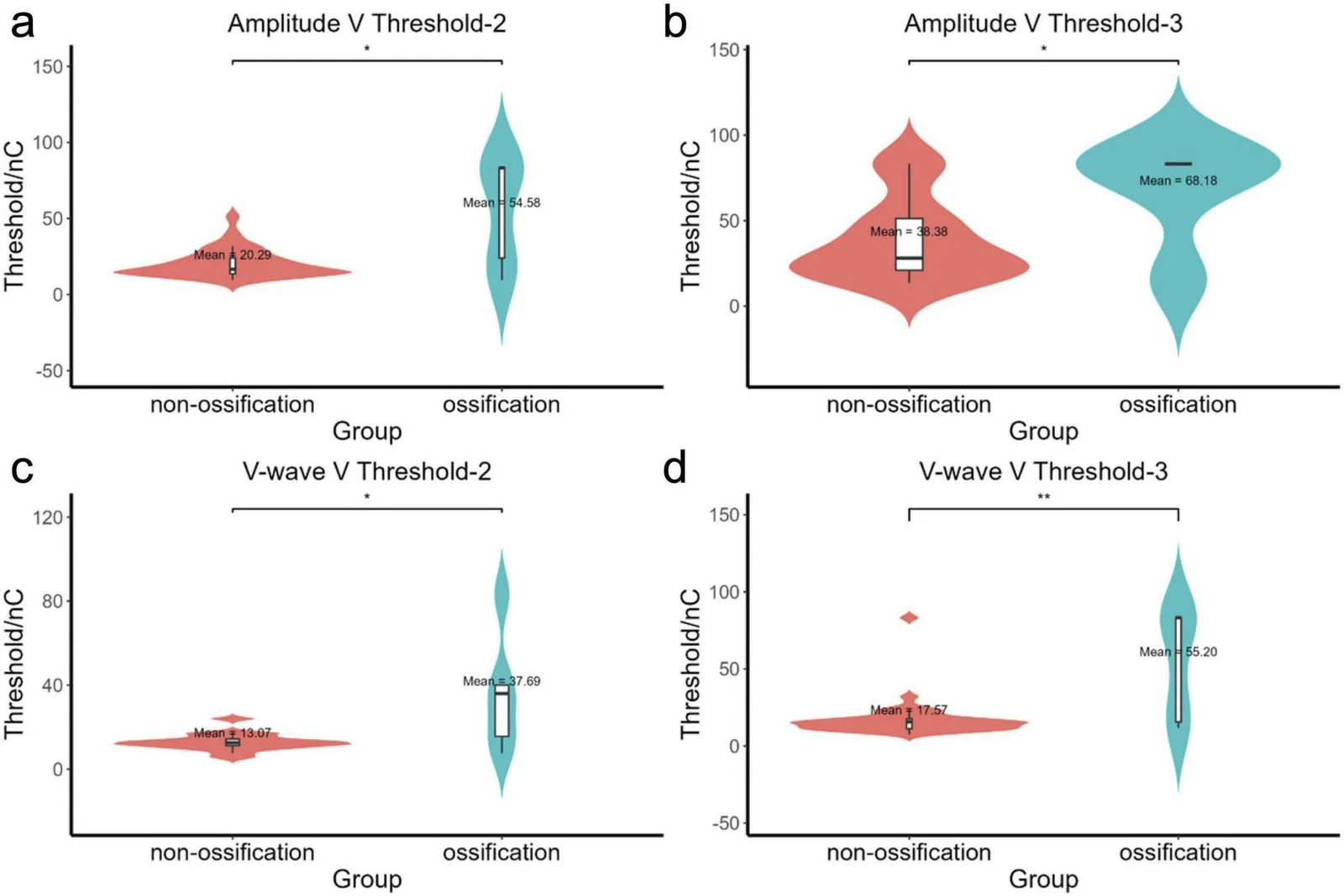
V-wave thresholds, including both the 0.5-μV threshold **(a,b)** and the disappearance threshold **(c,d)**, were significantly lower at the middle and basal electrodes in the non-ossification group compared with the ossification group. *p < 0.05, **p < 0.01.

**TABLE 3:** Comparison of evoked auditory brainstem response (EABR) measures and speech and auditory performance between the non-ossification and ossification groups.

EABR measures	*P*-value	Statistical value
Gibson–apical	0.002298	N/A
Gibson–middle	0.001532	N/A
Gibson–basal	0.0001436	N/A
Apical-V latency/ms	0.8269	*t* = 0.2253
Middle-V latency/ms	0.6251	*t* = 0.5085
Basal-V latency/ms	0.2255	*t* = −1.3912
Apical-III latency/ms	0.1736	Z = 1.382, U = 57.5
Middle-III latency/ms	0.8316	Z = 0.234, U = 84.5
Basal-III latency/ms	0.3769	Z = 0.91, U = 43
V threshold 0.5–apical/nC	0.07189	Z = −1.816, U = 207.5
V threshold-0.5–middle/nC	0.02899	Z = −2.2, U = 200.5
V threshold-0.5–basal/nC	0.03364	Z = −2.141, U = 198
V threshold-apical/nC	0.07779	Z = −1.779, U = 206.5
V threshold-middle/nC	0.01236	Z = −2.518, U = 210
V threshold-basal/nC	0.00836	Z = −2.654, U = 214
V amplitude–apical	0.1977	Z = 1.237, U = 68
V amplitude–middle	0.2237	Z = 1.084, U = 60
V amplitude–basal	0.2883	Z = 3.77, U = 45.5
CAP	0.0001735	Z = 3.921, U = 42
SIR	9.393e-05	Z = 3.603, U = 46
MONO	0.000333	Z = 3.638, U = 45
DSP	0.0002907	Z = −1.816, U = 207.5

Regarding auditory–speech performance, the ossification group demonstrated significantly poorer outcomes in CAP and SIR scores (Figures 4a, b), as well as lower monosyllabic and disyllabic word-recognition rates (Figures 4c, d), compared with the non-ossification group.

**FIGURE 4:**
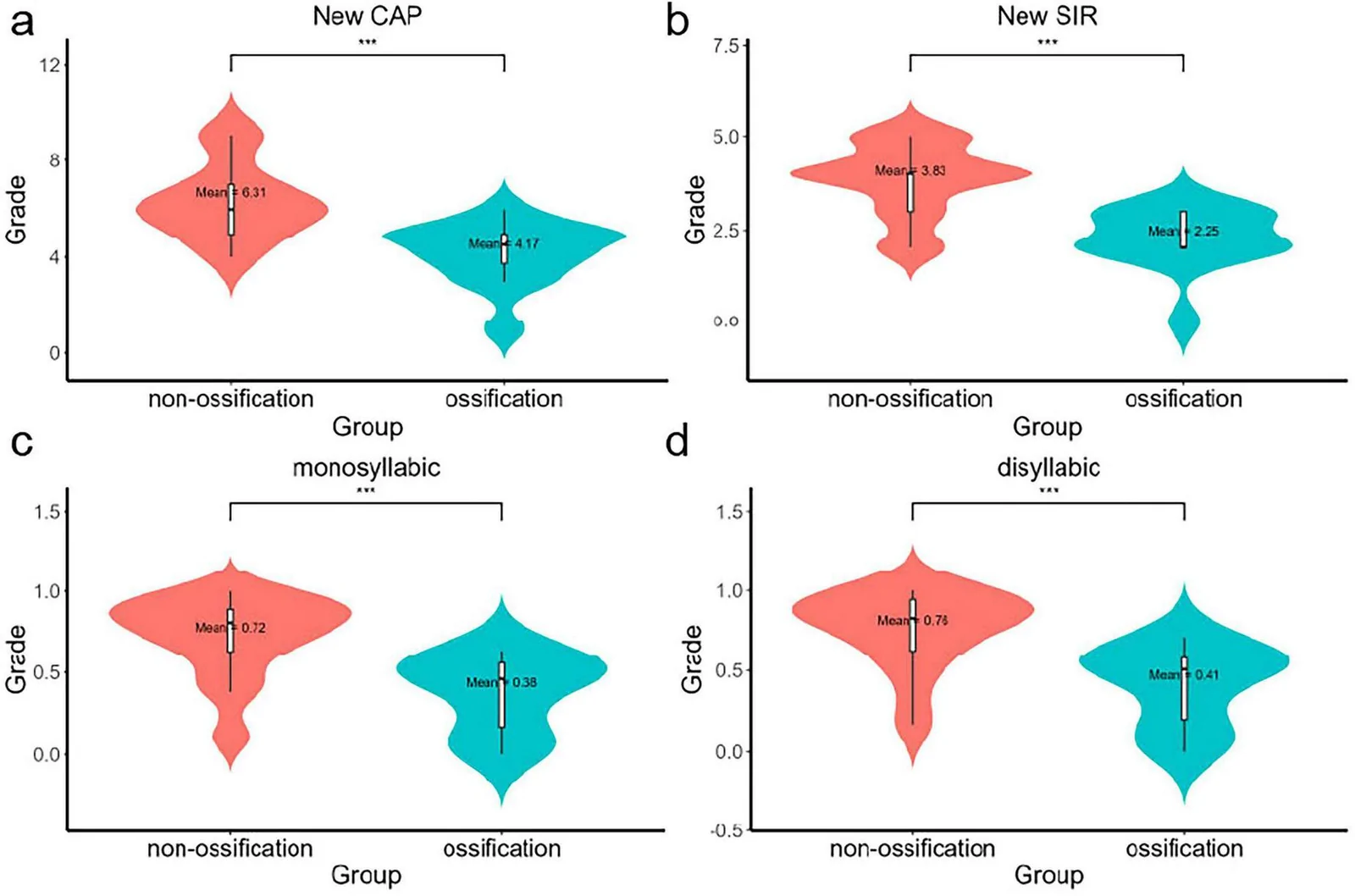
The ossification group demonstrated significantly poorer Categories of Auditory Performance (CAP) and Speech Intelligibility Rating (SIR) scores **(a,b)**, as well as lower monosyllabic and disyllabic word-recognition rates **(c,d)**, compared with the non-ossification group. ***p < 0.001.

### 3.3 Factors influencing postoperative auditory–speech performance following CRI

Univariate regression analysis demonstrated that implantation age; cochlear ossification; Gibson scores recorded at the apical, middle, and basal electrodes; and V-wave thresholds were significantly associated with auditory–speech performance outcomes, including CAP, SIR, and monosyllabic and disyllabic word-recognition scores. Detailed R^2^ and *p*-values are presented in Table 4. Among these variables, the Gibson score recorded at the middle electrode showed the strongest correlation with the disyllabic word-recognition rate (R^2^ = 0.4057, *p* = 0.0007).

**TABLE 4:** Univariate analysis of the correlation between evoked auditory brainstem response (EABR) parameters and auditory–speech performance.

	CAP			SIR			MONO			DSP		
Electrode/parameter	R^2^	F statistic	*P*-value	R^2^	F statistic	*P*-value	R^2^	F statistic	*P*-value	R^2^	F statistic	*P*-value
Implantation age	0.0994	4.1942	0.04752	0.113	4.8426	0.03392	0.1213	5.1082	0.02979	0.1449	6.2692	0.01682
Age difference	0.01377	0.5304	0.4709	0.006048	0.2312	0.6334	0.01992	0.7521	0.3914	0.02893	1.1022	0.3006
Apical III latency/ms	0.04277	1.4296	0.2406	0.03244	1.0728	0.3081	0.03007	0.9609	0.3345	0.02962	0.9461	0.3382
Middle III latency/ms	0.09113	3.2087	0.08271	0.08183	2.8521	0.101	0.06647	2.2074	0.1475	0.07206	2.4075	0.1309
Basal III latency/ms	0.01691	0.5161	0.4781	0.02289	0.7028	0.4085	0.02087	0.618	0.4382	0.02629	0.783	0.3835
Apical V latency/ms	0.09738	3.5603	0.068	0.06582	2.325	0.1368	0.09901	3.5165	0.06991	0.0934	3.2969	0.07879
Middle V latency/ms	0.1096	4.0635	0.05202	0.05753	2.0144	0.1652	0.08674	3.0393	0.09087	0.08525	2.9823	0.09382
Basal V latency/ms	0.0465	1.463	0.2359	0.01226	0.3723	0.5463	0.01922	0.5684	0.457	0.01762	0.5201	0.4766
V threshold 0.5-apical/nC	0.2073	9.4161	0.004072	0.1807	7.9418	0.007799	0.2501	11.6746	0.001621	0.2327	10.6147	0.002498
V threshold 0.5–middle/nC	0.208	9.1934	0.004552	0.162	6.7662	0.01352	0.2548	11.6265	0.001691	0.2344	10.4069	0.002775
V threshold 0.5–basal/nC	0.1543	6.3869	0.01617	0.1103	4.3399	0.0446	0.08691	3.2364	0.0809	0.09626	3.6215	0.06553
V threshold–apical/nC	0.2228	10.3209	0.002771	0.2034	9.1948	0.004481	0.2694	12.9036	0.0009972	0.2547	11.9619	0.001445
V Threshold–Middle/nC	0.2243	10.122	0.003067	0.2207	9.9096	0.003353	0.2702	12.5883	0.001157	0.2532	11.5268	0.00176
V threshold–basal/nC	0.2932	14.5215	0.0005378	0.2126	9.4512	0.004075	0.3497	18.2837	0.0001458	0.3041	14.8611	0.0004897
V amplitude–apical	0.00004989	0.0016	0.9684	0.001806	0.0579	0.8114	0.001455	0.0452	0.8331	0.002955	0.0919	0.7638
V amplitude–middle	0.0004441	0.0138	0.9073	0.009331	0.292	0.5928	0.01078	0.3268	0.5718	0.01142	0.3467	0.5604
V amplitude–basal	0.002991	0.09	0.7662	0.004422	0.1332	0.7177	0.01259	0.3698	0.5479	0.01001	0.2933	0.5923
Gibson-apical	0.2805	6.8234	0.003146	0.2359	5.4041	0.009009	0.3205	8.0177	0.001404	0.3283	8.3108	0.001152
Gibson–middle	0.2989	4.69	0.007772	0.2609	3.8832	0.01755	0.3956	6.9829	0.0009599	0.4057	7.2805	0.0007426
Gibson–basal	0.2902	6.9518	0.002944	0.2448	5.5112	0.00845	0.3896	10.5333	0.0002898	0.3712	9.7422	0.0004731
Cochlear ossification	0.3174	18.1365	0.0001254	0.3805	23.9574	0.00001745	0.3049	16.671	0.0002206	0.3032	16.5345	0.0002319

CAP, Categories of Auditory Performance; SIR, Speech Intelligibility Rating; MONO, monosyllabic word recognition; DSP, disyllabic word recognition.

In the regression model adjusted for implantation age and cochlear ossification, several EABR parameters remained significantly associated with auditory–speech outcomes. Specifically, Wave III latency recorded at the apical and middle electrodes, as well as Wave V latency at the apical electrode, demonstrated significant negative correlations with CAP scores. In addition, Wave III latency at the apical and middle electrodes was significantly negatively correlated with SIR scores, whereas the Gibson score at the basal electrode showed a significant positive correlation with monosyllabic and disyllabic word-recognition rates. These findings are summarized in Table 5.

**TABLE 5:** Multivariate regression analysis of the correlation between EABR parameters and auditory–speech performance (covariates: implantation age + presence of ossification).

	CAP						SIR					
Outcome measure	R^2^	F statistic	*P*- value	β	T-value	β_*P*-value	R^2^	F statistic	*P*-value	β	T-value	β_*P*-value
Apical III latency/ms	0.4995	9.9795	0.0001011	−1.18432407	−2.1583	0.039035	0.5977	14.8576	0.000004134	−0.78731745	−2.3529	0.0253764
Middle III latency/ms	0.5001	10.0051	0.00009921	−1.48421797	−2.2965	0.028804	0.5913	14.4674	0.000005216	−0.93814894	−2.4361	0.0209961
Basal III latency/ms	0.4115	6.5258	0.001738	−0.73377312	−1.2052	0.238208	0.5415	11.0229	0.00005946	−0.61125262	−1.6859	0.102935
Apical–V latency/ms	0.5312	11.7068	0.00002708	−0.88517617	−2.0998	0.0439789	0.5938	15.105	0.000003084	−0.45689601	−1.7485	0.0902767
Middle–V latency/ms	0.5099	10.7498	0.00005291	−0.77146085	−1.7727	0.0861087	0.5573	13.0074	0.00001138	−0.27262809	−1.0173	0.316863
Basal–V latency/ms	0.4148	6.6153	0.001612	−0.584631	−1.2723	0.213746	0.5044	9.4998	0.0001712	−0.20856143	−0.731	0.470851
V threshold 0.5–apical/nC	0.4828	10.5808	0.00004604	−0.00864735	−0.7105	0.482216	0.5394	13.2724	0.000006753	−0.00064656	−0.0843	0.933332
V threshold 0.5–middle/nC	0.4716	9.8157	0.00008922	−0.00837469	−0.6729	0.505715	0.522	12.0146	0.00001781	0.00123529	0.1573	0.87598
V threshold 0.5–basal/nC	0.4767	10.0201	0.0000763	−0.00801833	−0.8839	0.383151	0.5221	12.0186	0.00001776	−0.0010035	−0.1745	0.86257
V threshold–apical/nC	0.5021	11.427	0.00002461	−0.01761493	−1.3554	0.184212	0.5509	13.903	0.000004434	−0.00772767	−0.9374	0.355186
V threshold–middle/nC	0.4724	9.8472	0.00008709	-0.01169985	−0.7095	0.483	0.5242	12.1212	0.00001653	−0.00437916	−0.4215	0.676131
V Threshold-Basal/nC	0.4836	10.3009	0.00006169	−0.012794	−1.1102	0.274945	0.5217	11.9989	0.000018	0.00033711	0.0458	0.963738
V amplitude-apical	0.477	9.1197	0.0001916	−0.35143753	−1.2359	0.226084	0.5766	13.6164	0.00000877	−0.31775199	−1.8554	0.0733836
V amplitude-middle	0.4293	7.2705	0.0008839	−0.13234147	−0.478	0.636208	0.5196	10.4563	0.00007895	−0.20219433	−1.2021	0.239055
V amplitude-basal	0.3954	6.1044	0.002492	−0.35193748	−0.8102	0.424686	0.4791	8.5854	0.000336	−0.26975604	−0.9866	0.332298
Gibson-apical	0.5183	8.8752	0.00005579	0.12083285	0.0792	0.937327	0.5558	10.3213	0.00001559	0.41617914	0.4254	0.673286
Gibson–middle	0.5093	6.4339	0.0003299	−0.15248494	−0.0917	0.927523	0.5378	7.2142	0.0001401	0.39929605	0.3729	0.711745
Gibson–basal	0.4826	7.4621	0.00023	0.88835299	0.9236	0.362631	0.5228	8.7638	0.00006776	0.16610318	0.271	0.78814
		**MONO**						**DSP**					
**R^2^**		**F statistic**	***P*-value**	**β**	***T*-value**	**β_*P*-value**	**R^2^**	**F statistic**	***P*-value**	**β**	***T*-value**	**β_*P*-value**
0.4551		8.0744	0.0004628	−0.15061684	−1.6442	0.110944	0.5339	11.074	0.00005155	−0.14513666	−1.7372	0.0929644
0.3946		6.3014	0.002003	−0.19009314	−1.7113	0.0976944	0.4607	8.2577	0.0004008	−0.19000723	−1.8263	0.0781182
0.3166		4.1686	0.01505	−0.09793996	−1.0449	0.305353	0.4302	6.7957	0.001469	−0.10908196	−1.2822	0.210676
0.4942		9.772	0.0001177	−0.12416034	−1.6442	0.110565	0.5601	12.7307	0.00001536	−0.11049361	−1.5868	0.123036
0.4185		7.1956	0.0008881	−0.09424085	−1.228	0.22899	0.4745	9.0279	0.0002054	−0.08444607	−1.1644	0.25344
0.2921		3.7139	0.02341	−0.02599792	−0.3489	0.729873	0.3965	5.9141	0.00308	−0.0145403	−0.2126	0.833225
0.4923		10.6652	0.00004701	−0.0022469	−1.1627	0.253294	0.5223	12.028	0.00001764	−0.00179457	−0.956	0.34601
0.4525		8.8144	0.0002097	−0.00250596	−1.2878	0.207064	0.4815	9.9073	0.00009002	−0.00197356	−1.0403	0.306001
0.4247		7.8735	0.0004503	−0.00026652	−0.181	0.857483	0.4652	9.2766	0.0001459	−0.0003712	−0.261	0.795747
0.5208		11.9568	0.00001854	−0.00377314	−1.8439	0.0741952	0.5493	13.407	0.000006912	−0.00341054	−1.7161	0.0955225
0.453		8.8349	0.0002063	−0.00335091	−1.3015	0.202385	0.4822	9.9325	0.00008833	−0.00265945	−1.0596	0.297254
0.4773		9.7391	0.0001023	−0.00320923	−1.8044	0.0805828	0.4896	10.2306	0.00007066	−0.00222871	−1.2658	0.214736
0.4503		7.9177	0.0005239	−0.03838697	−0.8036	0.428192	0.5136	10.2057	0.0000942	−0.03287671	−0.7456	0.461887
0.3943		6.0766	0.002552	−0.03933264	−0.8992	0.376191	0.4677	8.1999	0.0004508	−0.03766666	−0.9397	0.355398
0.3552		4.9578	0.007193	−0.06706934	−0.9693	0.341007	0.4368	6.9815	0.001262	−0.06211509	−0.988	0.331912
0.5342		9.1758	0.00004684	0.04487951	0.1867	0.853049	0.5726	10.719	0.00001259	0.05099904	0.2212	0.826336
0.5473		7.2526	0.0001482	−0.06196578	−0.2528	0.802138	0.5915	8.6878	0.00003517	−0.06673182	−0.2861	0.776779
0.5084		8.0152	0.0001473	0.33190436	2.306	0.0279646	0.5336	8.8668	0.00006805	0.30016007	2.137	0.040594

CAP, Categories of Auditory Performance; SIR, Speech Intelligibility Rating; MONO, monosyllabic word recognition; DSP, disyllabic word recognition.

## 4 Discussion

In this study, we used EABRs to evaluate auditory brainstem pathway function in patients undergoing CRI. In patients with a normal cochlear anatomy, EABRs typically demonstrated a well-defined Wave V with normal latency, reflecting synchronous neural conduction through the auditory brainstem pathways, particularly the lateral lemniscus and inferior colliculus. In contrast, patients with significant cochlear ossification exhibited lower EABR elicitation rates, poorer waveform differentiation reflected by lower Gibson scores, and higher V-wave thresholds than patients without ossification. These findings may be explained by the fact that cochlear ossification impedes smooth insertion of the electrode array, reduces the number of effectively stimulated neural populations, and disrupts neural synchrony. Consequently, higher stimulation levels are required, resulting in broader or delayed neural activation caused by weak and desynchronized input to the brainstem. Furthermore, our findings demonstrate that pre-implantation EABRs recorded via the round window niche serve as a valuable localization tool during CRI surgery, particularly in cases with abnormal cochlear anatomy or ossification. However, no significant differences were observed in EABR latencies or V-wave amplitudes between the two groups. Cochlear ossification had a significant negative impact on postoperative auditory–speech performance. EABR thresholds and Gibson scores were significantly associated with postoperative outcomes, while latency and waveform characteristics also provided prognostic information. Collectively, our electrophysiological findings imply that EABRs, as an objective assessment of auditory brainstem pathway integrity, can facilitate successful execution of complex CRI procedures, support minimally invasive surgical approaches, and provide valuable prognostic information. These findings demonstrate the potential role of EABRs as an important adjunct for optimizing surgical planning and improving outcomes in challenging CRI cases.

Fibrous tissue formation and neo-osteogenesis have been documented in histopathological studies of temporal bone specimens (Kamakura and Nadol, 2016). Kamakura and Nadol (2016) reported that postoperative CNC word scores were negatively correlated with the volume of newly formed bone within the scala tympani, scala media/vestibuli, and the cochlea. In our ossification group, patients demonstrated substantial ossification obstructing the basal turn insertion pathway, which likely altered electrical stimulation patterns around the electrode array. This observation is consistent with our findings of elevated EABR thresholds and poorer Gibson scores in the ossification group. Ossified tissue and fibrous bands may reduce the efficiency of the electrode–neural interface, leading to increased auditory thresholds and reduced dynamic ranges. Among the EABR parameters evaluated, the Gibson score demonstrated the strongest predictive value, particularly for mid- to high-frequency auditory regions. Although ossification significantly affected waveform elicitation, EABR latencies—which may reflect neural plasticity and maturation—remained relatively stable in CRI patients. Consequently, the Gibson score, which reflects the overall differentiation of Waves III and V, appeared to be the most suitable parameter for predicting postoperative outcomes following CRI.

In recent years, clinical attention to EABRs has declined, largely because of the widespread adoption of electrically evoked compound action potentials (ECAPs), which provide rapid, convenient, and universally applicable testing. ECAPs can provide comprehensive electrode-specific data for predicting postoperative outcomes and determining T- and C-levels during dynamic range mapping (Antony et al., 2025), gradually overshadowing the advantages of EABRs in primary cochlear implantation. However, in CRI surgery, both our findings and reports from other centers indicate that complex conditions such as cochlear fibrosis, ossification, and hyperplastic tissue formation are frequently encountered (Donati et al., 2023, Kamakura and Nadol, 2016). CRI is often performed after medical complications, including meningitis, flap necrosis, infection, or severe pre-existing cochlear malformations. Bacterial infection, foreign-body reactions to electrode materials, bone dust, or blood contamination may promote neo-ossification, resulting in insertion difficulties and uncertain electrode positioning (Kamakura and Nadol, 2016). Under these conditions, conventional postoperative ECAPs cannot provide pre-implantation localization or prediction of neural responsiveness because of the disruption of the normal cochlear architecture. In our study, Patient No. 6 in the ossification group presented with severe ossification of the basal turn, making identification of the cochlear insertion pathway difficult after bone removal. EABR testing was used to confirm the appropriate insertion site, allowing partial electrode insertion (18/24 electrodes). This case illustrates that pre-implantation EABRs enable assessment of neural responsiveness directly at the drilling site and may provide important guidance for electrode reimplantation.

Previous studies have demonstrated that most patients undergoing CRI achieve postoperative outcomes comparable to or better than those obtained after the initial implantation (Gawecki et al., 2016). In most cases, CRI permits successful electrode reinsertion, and residual hearing may even be preserved without additional cochlear trauma (Ikeya et al., 2013). However, perielectrode ossification can complicate CRI surgery by impairing complete electrode insertion and optimal positioning, thereby adversely affecting postoperative outcomes (Terry et al., 2015). In the present study, the ossification group demonstrated significantly poorer auditory–speech performance than the non-ossification group (CAP: *p* = 0.00017; SIR: *p* < 0.0001; monosyllabic recognition: *p* = 0.00033; disyllabic recognition: *p* = 0.00029), indicating that cochlear ossification/fibrosis and the ability to achieve complete electrode insertion are critical determinants of CI outcomes. Importantly, Gibson scores and EABR V-wave thresholds demonstrated the strongest correlations with postoperative outcomes. The Gibson score is used to evaluate the quality and configuration of EABR waveforms, particularly Waves III and V, with higher scores indicating more robust and synchronized neural responses along the auditory pathway. In addition, the input/output slope reflects neural recruitment and synchrony (Chen et al., 2024). The Gibson score recorded at the middle electrode demonstrated the strongest correlation with the disyllabic word-recognition rate (R^2^ = 0.4057, *p* = 0.0007). Furthermore, in multivariate models adjusted for implantation age and cochlear ossification, Wave III latencies at the apical and middle electrodes showed significant negative correlations with CAP and SIR scores, whereas the Gibson score at the basal electrode demonstrated significant positive correlations with monosyllabic and disyllabic word-recognition rates (Table 5).

Because the human cochlea is tonotopically organized, the middle electrode region generally corresponds to frequencies between 1,000 and 2,000 Hz, which are critically important for encoding disyllabic speech sounds. In contrast, the basal turn encodes higher frequencies (approximately 2,000–8,000 Hz), which contribute disproportionately to several aspects of speech perception, including consonant identification, discrimination of voiceless fricatives and affricates, and temporal envelope processing. Therefore, favorable basal-turn electrode electrophysiological characteristics may indicate superior monosyllabic and disyllabic word-recognition performance, particularly under noisy listening conditions. Meanwhile, the middle electrode region may represent a “transition zone” between relatively preserved apical neural structures and the more vulnerable basal turn. A high Gibson score at the middle electrode may therefore reflect better preservation of neural synchrony and intact III–V interpeak intervals, indicating preserved temporal processing integrity. These mechanisms may explain the strong correlation between middle-array electrode parameters and postoperative disyllabic word-recognition performance. Our findings are consistent with those of Gallego et al., who reported that EABR Wave V latency and the III–V interpeak interval were associated with speech-recognition outcomes. Collectively, these findings imply that intraoperative and postoperative electrophysiological assessments are valuable for early prognostic evaluation and optimization of postoperative patient management.

Histological studies have demonstrated that round window insertion causes less inner-ear trauma and results in lower degrees of fibrosis and ossification than does cochleostomy (Bi et al., 2021). Minimally invasive surgical techniques, including low-speed cochleostomy, prevention of bone dust and blood contamination within the cochlea, and slow electrode insertion to avoid injury to the osseous spiral lamina and basilar membrane, may reduce cochlear ossification and fibrosis while preserving residual hearing. Somdas et al. (2007) suggested that cochlear ossification and fibrosis may extend from the apex toward the base, with the most severe ossification typically occurring in the basal turn. Similar findings were observed in our study, in which two cases of severe ossification demonstrated marked basal turn involvement. In Patient No. 6, partial electrode insertion was achieved by opening the basal turn and removing hyperplastic bone, thereby preserving a degree of auditory and speech function. Previous studies have also indicated that cochlear ossification and fibrosis may contribute to spiral ganglion cell loss, potentially affecting long-term CI outcomes.

## 5 Limitations

Several limitations of this study should be acknowledged. First, the relatively small sample size, particularly in the ossification group (*n* = 12), limits statistical power and precludes more robust subgroup analyses. Second, the 3-month follow-up period is relatively short; longer-term studies (6–12 months) are needed to confirm the stability of these outcomes. Third, missing EABR data for several patients, particularly in the ossification group, may have introduced selection bias. Fourth, the prognostic associations we observed are correlational; external validation in an independent cohort is necessary before these findings can be considered definitive. Future multicenter, prospective studies with larger sample sizes are needed to confirm and extend our results.

In conclusion, this study highlights the utility of EABR as an objective method for assessing auditory brainstem pathway integrity during CRI, particularly in patients with cochlear ossification or anatomical abnormalities. EABR parameters, including V-wave thresholds, Gibson scores, and waveform latencies, provide valuable prognostic information regarding postoperative auditory–speech outcomes. These findings support the integration of EABRs into surgical planning and intraoperative assessment in complex CRI cases to optimize electrode placement and improve patient prognosis.

## Data Availability

The datasets presented in this study can be found in online repositories. The names of the repository/repositories and accession number(s) can be found in the article/supplementary material.
